# Longitudinal relationship between 24-Hour Movement behavior patterns and physical function and quality of life after stroke: a latent transition analysis

**DOI:** 10.1186/s12966-024-01689-1

**Published:** 2024-12-18

**Authors:** Yi Zhang, Lin Yao, Lei Chen, Weiying Zhong, Jiaxuan Li, Lan Xu, Xi Pan

**Affiliations:** Nursing Department, The First Affiliated Hospital of Soochow University, 899 Pinghai Road, Suzhou, 215000 Jiangsu China

**Keywords:** Physical activity (PA), Sedentary behavior, 24-hour movement behaviors, Stroke, Latent transition analysis

## Abstract

**Background:**

24-hour movement behavior, including Physical activity (PA), Sedentary behavior (SB), and sleep, is independently associated with health after stroke. Few studies have explored 24-hour movement behavior patterns in stroke survivors and their transitions, as well as the health implications of the transitions. This study aimed to explore the different subgroups and stability of 24-hour movement behavior patterns in people after stroke and the relationship of profile transitions with physical function and health-related quality of life (HRQoL).

**Methods:**

In this study, 131 people with first-ever stroke were investigated at one week (T1), one month (T2), three months (T3), and six months (T4) after discharge. The participants were asked to wear a wristband smartwatch for 7 consecutive days during each pe riod to collect 24-hour exercise data. After each period, their physical function and HRQoL were assessed. Latent profile analysis (LPA) identified typologies of 24-hour movement behaviors, and latent transition analysis (LTA) examined the stability and change in these profiles over time. The relationship of transition types with physical function and HRQoL was analyzed using a generalized linear regression model.

**Results:**

108 participants were categorized into 3 latent profiles of 24-hour movement behavior: “Active, Non-sedentary, and Short sleep,” “Active and Sedentary,” and “Inactive and Sedentary.” The LTA results indicated that the proportion of participants with the “Active, Non-sedentary, and Short Sleep” profile and “Active and Sedentary” profile staying in the original latent profile was high. However, participants in the “Inactive and Sedentary” profile showed a high probability of transitioning to “Active and Sedentary” profile (T1→T2: 65.2%; T2→T3: 76.3%; T3→T4: 51.7%;T1→T4: 54.2%). Transition types are associated with physical function and HRQoL.

**Conclusions:**

The results demonstrated substantial transitions in 24-hour movement behaviors within 6 months of rehabilitation after discharge, associated with later physical function and HRQoL. Furthermore, the participants’ sedentary behavior was highly stable within 24-hour movement behaviors, necessitating prompt diagnosis and intervention.

**Supplementary Information:**

The online version contains supplementary material available at 10.1186/s12966-024-01689-1.

## Background

According to the Global Burden of Disease Study [1], stroke is the second leading cause of death and the third leading cause of disability worldwide. There are reportedly more than 80 million people living with stroke worldwide [2]. International guidelines suggest that people with stroke should engage in at least 150 min of moderate-intensity physical activity (PA) per week [3]. People after stroke are also recommended to reduce sedentary behavior (SB), although evidence is insufficient to quantify an SB threshold [3]. However, people after stroke often grapple with varying degrees of physical and psychosocial sequels, such as dyskinesia [4], post-stroke fatigue [5], and depression [6], which can result in physical inactivity. A significant proportion of people after stroke fail to meet guidelines and their daily steps are lower than age-matched controls [7]. Moreover, people after stroke tend to spend more time in the sedentary state compared to their age-matched peers [8]. Studies have shown that PA has the potential to influence functional recovery, cardiometabolic health and health-related quality of life (HRQoL) in people after stroke [9–13]. Sedentary behavior also associated with cardiovascular health in people after stroke [14]. Additionally, people typically experience sleep disorders after stroke, with 40% of them reporting insomnia [15, 16]. Post-stroke sleep disorders are also associated with poor functional outcomes and quality of life [15]. However, these studies have mainly dealt with the independent relationship between a specific health outcome in people after stroke and any one of PA, SB and sleep. They have failed to acknowledge that individuals have a certain and unchanging amount of time in a day, and any changes in the time allocated to one behavior will unavoidably affect the time spent on other behaviors [16]. There are limitations in considering the effect of either behavior of the 24-hour cycle on health in isolation. A new term, “24-hour movement behaviors”, which includes PA, SB, and sleep, has been recently agreed upon internationally. As a result, some countries have revised 24-hour movement guidelines to encompass a full day’s movement behaviors [17–19].

However, few studies have explored 24-hour movement behaviors in people after stroke under this perspective. Wondergem et al. conducted a study using accelerometer to investigate the movement behavior of people after stroke during waking time 2 weeks after discharge. They identified three patterns of movement behaviors: sedentary exercisers, sedentary movers, and sedentary prolongers [20]. Then, they conducted a two-year longitudinal study and found that patterns of movement behavior 2 weeks after discharge were associated with physical function [21], and 35% of participants’ movement behavior patterns changed within the first two years, of whom 63% deteriorated to a movement behavior pattern with higher health risks [22]. This suggests that movement behavior patterns in people after stroke are associated with health outcomes, and their patterns of movement behavior may deteriorate over time. However, these studies only investigated movement behaviors during wakefulness in people with stroke, ignoring sleep as a specific form of activity. Moreover, they did not explore the relationship between transitions in movement behavior patterns and health. Little is known about the association between transitions in 24-hour movement behavior patterns in people after stroke and health outcomes.

Latent profile analysis (LPA) is a person-centered analysis that focuses on the heterogeneity of individual responses, classifying individuals into common profiles to identify different subgroups [23]. LPA has been widely used in recent years to identify cross-sectional patterns and longitudinal changes in movement behavior in different populations [24–26]. However, to the best of our knowledge, no previous study applied LPA to examine the types of 24-hour movement behaviors patterns among people after stroke. The adoption of the LPA approach allows for simultaneous examination of PA, SB, and sleep in people after stroke, rather than treating each behavior separately. This approach provides insights into how different subgroups of people after stroke allocate their time to PA, SB, and sleep, thereby enabling the development of targeted interventions. Furthermore, latent transition analysis (LTA), as an extension of LPA, can be employed to examine the transition patterns of the 24-hour movement behavior profile in people after stroke [27]. By understanding the evolution of patients’ 24-hour movement behaviors, we can better predict their recovery trajectories and tailor prevention and intervention strategies. This approach was particularly valuable in our study, as we aimed to (1) identify the different latent profile types of 24-hour movement behaviors in people after stroke after discharge; (2) explore the stability of different latent profiles over time; (3) explore the relationship of transitions in 24-hour movement behavior profiles with physical functioning and HRQoL.

## Methods

### Participants and study design

This was a prospective, longitudinal study of 24-hour movement behaviors in people after stroke. The study was approved by the Medical Ethics Research Committee of the First Affiliated Hospital of Soochow University (No.2022518). The participants were selected from 517 patients with stroke who visited the authors’ neurology department from September 2022 to June 2023. The inclusion criteria were as follows: clearly diagnosed first-ever stroke; aged ≥ 18 years old; able to get out of bed independently; normal language conversation and comprehension; the participant or their family members must have a smartphone and know how to use WeChat; and willingness to participate in this study. The exclusion criteria included: affliction with severe cardiac, pulmonary, neurological, musculoskeletal, or psychiatric disorders; unfit to participate in the study due to therapeutic and other factors; and severe sequelae and comorbidities (e.g., heart failure). Participants were recruited if they met all inclusion criteria and had none of the exclusion criteria.

First, 140 people after stroke who met the criteria were recruited, and finally 131 patients consented to participate in this study. The participants filled out a demographics form before discharge. Then, they were surveyed at home one week (T1), one month (T2), three months(T3), and six months (T4) after discharge. These four time points were selected based on the stroke recovery trajectory, encompassing trajectory onset, initial rehabilitation, continued rehabilitation, and semi-stable phase [28]. The participants were instructed to wear a wristband smartwatch continuously for 7 consecutive days during each survey period, except when they were charging the device or when they were taking a bath and swimming) [29]. At the end of each survey period, the physical function and HLQoL of participants were assessed by completing the Modified Rankin Scale (mRS) and EuroQol Five-Dimensions Five-Level Questionnaire (EQ-5D-5 L) via telephone calls.

## Methods

### Participants’ characteristics

The authors developed a questionnaire based on the characteristics of patients with stroke, which consisted of sociodemographic and disease-related information. Sociodemographic information included age (years), gender, body mass index (BMI, kg/m^2^), occupation, smoking history, drinking history, educational attainment, marital status, place of residence, residential status, family per capita monthly income (yuan), and medical insurance. Height and weight were objectively measured to calculate body mass index, and other information was self-reported.

Age was categorized into the < 65 years and ≥ 65 years groups [30]. BMI was categorized into underweight (BMI < 18.5), normal weight (18.5 ≤ BMI <25), and overweight/obese (BMI ≥ 25) [31]. Patients with a history of smoking (smokers) were defined as those who smoked at least one cigarette per day for one year [32]. People with a history of drinking were defined as patients with a history of alcohol intake ≥ 20 g/d for more than 3 years [33]. Education attainment was categorized into elementary school and below, high school/secondary school, post-secondary, and college and above. Marital status was categorized into married and unmarried/divorced/widowed. Place of residence was categorized into rural, county seat, or urban. Residential status was categorized into living alone or living with family. Family per capita monthly income, equal to family monthly income divided by the number of family members, was categorized as <3000, 3000–5000, and >5000. Medical insurance was categorized as urban employee basic medical insurance, urban and rural resident basic medical insurance, and self-financed. Disease-related information, including comorbid chronic disease, days in hospital severity of stroke, physical function recovery and ability to perform activities of daily living (ADL), was obtained from medical records.

A summary comorbidity variable was created based on the presence of hypertension, diabetes mellitus, coronary heart disease, and other chronic diseases. We stratified the hospitalization length into ≤10 days group and>10 days group based on average days in hospital for people with stroke in China [34]. National Institute of Health Stroke Scale (NIHSS) was used to measure the severity of stroke [35]; the total score on this scale ranges between 0 and 42, and higher scores indicate a more severe stroke. Scores 0–4, 5–12, 16–20, and more than 20 represent mild, moderate, moderate to severe, and severe stroke, respectively. In this study, the participants were grouped into two categories: mild (≤ 4) or non-mild (>4). ADL was assessed using the Barthel Index (BI) [36]. The total BI scores can range from 0 to 100, and higher scores indicate higher levels of ADL independence. A BI score of < 40, 40–60, 60–99, and 100 indicates severe dependence, moderate dependence, mild dependence, and no dependence, respectively. In this study, the participants were grouped into two categories: moderate to severe dependence (≤ 60) or mild to no dependence (>60).

## Movement behaviors

Data on 24-hour movement behaviors were collected using a smartwatch (VZVK-E80), which was manufactured by a company in Shenzhen, Guangdong Province, China. The smartwatch contained built-in gyroscope, accelerometer, and photoplethysmography sensor, collecting data including daily steps, active hours, sedentary hours, sleep hours, vital signs, and other indicators. Accelerometers exhibited exceptional reliability and precision in measuring movement behavior [37]. Before starting the study, we undertook rigorous calibration and validation procedures to ascertain the device’s accuracy specifically for people after stroke. We evaluated the accuracy of the device across various arm swing conditions in a cohort of 51 people after stroke, comparing it with video monitoring (regarded as the gold standard). The test was conducted during hospitalization and participants wore smartwatch on the left and right wrist and completed a 3-minute walk on a flat surface with different arm swings (free arm swing, restricted arm swing, restricted right arm swing, and restricted left arm swing), respectively. The walking test was repeated the next day with different swing arm modes for repetitive analysis. Compared to video monitoring under conditions of free arm swing, the smartwatch demonstrated an error rate of 2.26%. Moreover, 10 people after stroke wore smartwatches for 24 h and recorded their sleep throughout the day using sleep diaries. The error rate of sleep duration measured by smartwatch was within 5% compared to a paper diary.

Upon discharge, the participants were given instructions by the researchers regarding the correct way to wear and utilize the device. All participants wore the same model smartwatch (VZVK-E80). During the follow-up period, researchers utilized phone calls or WeChat messages to prompt participants to wear their devices. Before and after each stage, researchers proactively communicated with the participants via telephone to distribute and collect devices through mail. Non-wear time intervals were defined as more than 30 consecutive minutes without wearing the device. Researchers verified the wearing time of participants every day at 8:00 PM. For those who wore the device for less than 24 h, researchers conducted follow-up assessment via phone or WeChat regarding their movement behavior during the period when the device was not worn. This ensured that any missing data were accurately filled in, and our dataset comprehensively reflected the participant’s distribution of movement behavior across a full 24-hour cycle. The minimum wear time was determined to be ≥ 3 days and ≥ 8 h per day [38]. Participants were included in subsequent analyses if they met this criterion.

## Modified Rankin Scale

The Modified Rankin Scale was used to assess the degree of physical function recovery in people after stroke [39]. The scale comprises six items that classify the patient’s neurological functioning level on a scale of 1 to 6, with scores ranging from 0 to 5. Scores 0, 1, 2, 3, 4, and 5 indicate no symptoms at all, no significant functional impairment despite symptoms, mild disability, moderate disability, moderate to severe disability, and severe disability, respectively; higher scores indicate a lower level of physical function recovery.

### EuroQol five-dimensions five-level

The EQ-5D-5 L was used to measure the HRQoL of the participants. It is a set of standardized scales to measure health status, consisting of a short descriptive system and a thermometer-like visual analog scale (VAS) [40]. The descriptive system comprises five dimensions: mobility (MO), self-care (SC), usual activities (UA), pain/discomfort (PD), and anxiety/depression (AD). Each dimension includes five options: no, slight, moderate, severe, and extreme problems/unable. In this study, the EQ-5D health utility values were calculated using a utility value point system developed for Chinese populations [41], with higher health utility values representing higher levels of HRQoL.

### Data analysis

All statistical analyses were performed in SPSS 25.0 and Mplus 8.2. Qualitative data were described using frequency and percentage. Differences in baseline information between included and excluded participants were compared by chi-square and Fisher’s exact test.

The mean PA, SB, and sleep per day were derived for each participant. Participants wore the device for varying durations, depending on the day of the week when the data was collected. The data on the PA, SB, and sleep of participants were analyzed using LPA to identify subgroups of 24-hour movement behavior patterns. The selection of the best LPA solution usually depends on the model fit indicators. The model fit indicators mainly include the Akaike Information Criterion (AIC), the Bayesian Information Criterion (BIC), the adjusted Bayesian information criterion (ABIC), Entropy, the adjusted Vuong-Lo-Mendell-Rubin likelihood ratio test (A-LRT), and the bootstrap likelihood ratio test (BLRT). Smaller values of AIC, BIC, and ABIC indicate better model fit. An entropy value greater than 0.80 indicates good clarity of classification [42]. The difference in fit between the K-1 and K- cluster models is determined by the p-value of the A-LRT and BLRT indicators. A significant p-value indicates that the fitting of the K-cluster model is better than that of the K-1 cluster model [43]. The minimum group size was set at 5% of the total sample to ensure representative profile samples [44]. LTA is an extended longitudinal LPA model, which can be used to explore the probabilities of transitions between latent statuses over time [27]. The transition probability refers to the likelihood that people after stroke transitioned to, or stayed in, one latent status from T1 to T2, T2 to T3, T3 to T4 and T1 to T4. Mplus 8.2 was used to conduct LPA and LTA.

We used chi-square and Fisher’s exact test to investigate whether there were differences between profiles in terms of socio-demographic information. Differences in socio-demographic information between transition patterns were also examined. Furthermore, generalized linear regression models were implemented to examine the relationship of transitions in 24-hour movement behavior profiles with the follow-up physical function and HRQoL. The reference was someone who continued to remain in the same profile over time. Age, gender, smoking, and alcohol history were all controlled in these models. A two-tailed *P*-value < 0.05 was represented as statistical significance.

## Results

**Fig. 1:**
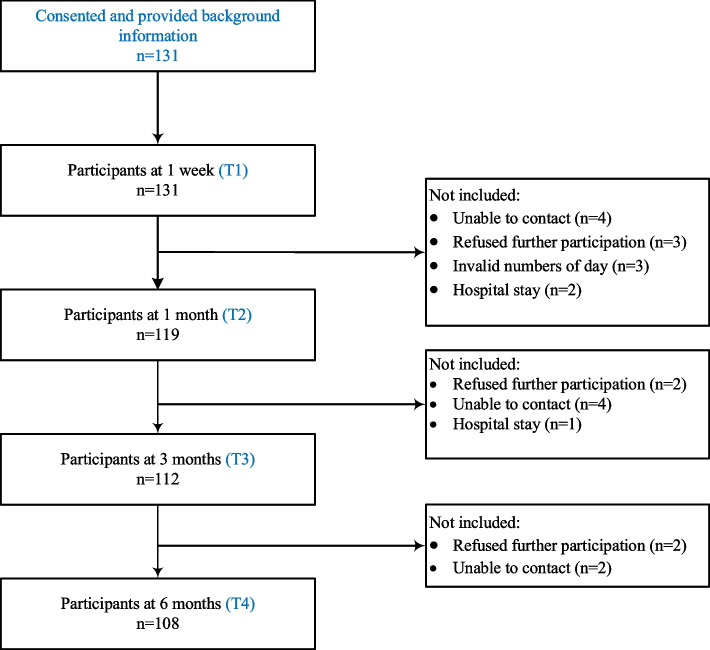
Study flowchart

In total, 131 people after stroke consented to participate in this study. Of these, 108 (82.4%) completed the surveys at four time points: one week, one month, three months and six months (flowchart and reasons for refusal are presented in Fig. 1). The data showed that 61 participants (56.48%) were under 65 years old and 67 of them (62.04%) were male. Table 1 presents the demographics of the participants. There was no statistical difference between the included and excluded participants in terms of demographics (*p* > 0.05).

Data from each of these participants met the minimum wear time criteria at each time point. The average wear time of the smartwatch and the average values for each behavior at each time are shown in Table 2.

**Table 1:** Comparison of baseline characteristics between the included and excluded participants n (%)

Characteristic	Included (*N* = 108)	Excluded (*N* = 23)	*X*^2^	*P*
Age (years)			0.149^a^	0.699
≤ 65	61 (56.48)	14 (60.87)		
> 65	47 (43.52)	9 (39.13)		
Gender			0.463^a^	0.496
Male	67 (62.04)	16 (69.57)		
Female	41 (37.96)	7 (30.43)		
BMI (kg/m^2^)			1.851^c^	0.395
< 18.5	3 (2.78)	1 (4.35)		
18.5–25	48 (44.44)	13 (56.5)		
≥ 25	57 (52.78)	9 (39.13)		
Occupation			0.128^a^	0.72
Employed	38 (35.19)	9 (39.13)		
Retirement/ Unemployed	70 (64.81)	14 (60.87)		
Smoking history			0.333^a^	0.564
Smoker	40 (37.04)	10 (43.48)		
Non-smoker	68 (62.96)	13 (56.52)		
Drinking history			0.072^a^	0.788
Drinker	36 (33.33)	7 (30.43)		
Non-drinker	72 (66.67)	16 (69.57)		
Education			1.210^c^	0.757
Elementary school and below	29 (26.85)	8 (34.78)		
High school/secondary school	48 (44.44)	11(47.83)		
Post-secondary	20 (18.52)	3 (13.04)		
College and above	11 (10.19)	1 (4.35)		
Marital status			0.001^b^	0.975
Married	97 (89.81)	20 (86.96)		
Unmarried/Divorced/Widowed	11 (10.19)	3 (13.04)		
Place of residence			4.195^c^	0.119
Rural	14 (12.96)	7 (30.43)		
County seat	5 (4.63)	0		
Urban	89 (82.41)	16 (69.57)		
Residential status			0.003^c^	0.716
Live alone	5 (4.63)	1 (4.35)		
Live with families	103 (95.37)	22 (95.65)		
Family per capita monthly income(yuan)			0.624^c^	0.686
< 3000	9 (8.33)	1 (4.35)		
3000 ~ 5000	74 (68.52)	18 (78.26)		
> 5000	25 (23.15)	4 (17.39)		
Medical insurance			0.686^c^	0.797
UEBMI	10 (9.26)	3 (13.04)		
URRBMI	96 (88.89)	20 (86.96)		
Self-financed	2 (1.85)	0		
Chronic disease			1.624^c^	0.627
0	3 (2.78)	0		
1	79(73.15)	17 (73.91)		
2	19 (17.59)	3 (13.04)		
≥ 3	7 (6.48)	3 (13.04)		
Days in hospital			0.583^a^	0.445
≤ 10	76 (70.37)	18 (78.26)		
> 10	32 (29.63)	5 (21.74)		
Physical function recovery (mRS)			0.708^a^	0.4
No to mild disability (0–2)	84 (77.78)	16 (69.57)		
Moderate to severe disability (>2)	24 (22.22)	7 (30.43)		
Stroke severity (NIHSS)			0.035^a^	0.851
Mild (≤ 4)	40 (37.04)	9 (39.13)		
Non-mild (> 4)	68 (62.96)	14 (60.87)		
Activities of daily living (BI)			1.168^b^	0.194
Mild to no dependence (> 60)	104 (96.30)	20 (86.96)		
Moderate to severe dependence (≤ 60)	4 (3.70)	3 (13.04)		

Notes: ^a^chi-square tests, ^b^Continuity Adjusted chi-square test, ^c^Fisher’s exact test

*BMI*, Body Mass Index calculated as weight in kilograms divided by height in meters squared, *UEBMI* Urban Employee Basic Medical Insurance, *URRBMI* Urban and Rural Resident Basic Medical Insurance, *mRS* Modified Rankin Scale, *NIHSS* National Institute of Health Stroke Scale, *BI* Barthel Index.

**Table 2:** Wear time and values of each behavior at each time point mean (standard deviation)

Time	Wear time	24-hour movement behavior		
		PA	SB	Sleep
T1	22.53(1.99)	8.12(3.13)	8.21(2.40)	7.67(1.25)
T2	21.96(3.16)	9.45(2.98)	7.32(2.28)	7.24(1.57)
T3	22.63(2.32)	9.84(2.39)	7.24(2.09)	6.92(1.10)
T4	22.48(2.28)	10.14(2.44)	6.77(2.15)	7.09(1.37)

### Profiles of 24-hour movement behaviors in people after stroke

The number of latent profiles for 24-hour movement behaviors increased progressively from 1 to 5 at each of the four points of time (Table 3). At T1, the AIC, BIC, and aBIC values of the 3-solution were smaller, the entropy was greater than 0.9, and the A-LRT and BLRT also suggested that the 3-solution was significantly better than the 2-solution. The AIC, BIC, and aBIC were smaller for the 4-solution, but the A-LRT in the 4-solution was not significant, suggesting that the fitting metrics for the 4-solution were not significantly improved compared to the 3-solution. As a result, the 3-solution was selected as the final solution at T1. At T2, AIC, BIC, and aBIC decreased with the increasing number of profiles. A-LRT revealed that the model fit was not significantly improved by including profiles in addition to the 2-solution. Conflicting model fit metrics are not uncommon; in these cases, it is recommended to assess the substantive significance and coherence of candidate models [45]. A closer inspection of the 3–5 solution showed that models containing more than 3 profiles produced classes that were similar to those already present, albeit to a slightly different extent. Therefore, the 3-solution was selected as the final solution at T2. Although A-LRT showed that the 2- and 4-solution fitted better at T3. BLRT demonstrated that an increased number of profiles resulted in a more optimal fit. Similar to the patterns identified at T2, a closer examination of the 3-, 4-, and 5-solution indicated that the new classes, beginning with the 3-profile model, exhibited a generally similar pattern of recognition to the existing classes. Considering the consistency, simplicity, and practicality of the model, the 3-solution was considered the final model at T3. A combination of all indicators at T4 suggested the 3-solution as the best model. The AIC, BIC, and aBIC values exhibited a decreasing trend as the number of profiles increased, suggesting an improvement in the model fit with the increasing number of profiles. The A-LRT analysis indicated that the 3-solution was not inferior to the 4-solution. Additionally, the entropy measure supported the notion that the 3-solution was superior to the 4-solution.

**Table 3:** Model fit of LPA of movement behavior

Time	Solution	AIC	BIC	aBIC	Entropy	A-LRT	BLRT
T1	1-solution	1412.819	1428.911	1409.953			
	2-solution	1303.208	1330.029	1298.432	0.825	0.0133	<0.001
	3-solution	1240.621	1278.171	1233.935	0.904	0.0113	<0.001
	4-solution	1217.43	1265.709	1208.834	0.908	0.1766	<0.001
	5-solution	1194.101	1253.108	1183.595	0.902	0.4375	<0.001
T2	1-solution	1439.01	1455.103	1436.144			
	2-solution	1362.745	1389.566	1357.969	0.918	0.0019	<0.001
	3-solution	1321.772	1359.321	1315.086	0.881	0.6106	<0.001
	4-solution	1294.175	1342.454	1285.579	0.856	0.0599	<0.001
	5-solution	1270.752	1329.759	1260.245	0.9	0.1741	<0.001
T3	1-solution	1296.281	1312.374	1293.416			
	2-solution	1212.957	1239.778	1208.181	0.789	0.013	<0.001
	3-solution	1169.369	1206.919	1162.683	0.825	0.2529	<0.001
	4-solution	1131.333	1179.611	1122.736	0.898	0.0337	<0.001
	5-solution	1106.842	1165.849	1096.336	0.929	0.0178	<0.001
T4	1-solution	1354.43	1370.523	1351.564			
	2-solution	1277.402	1304.223	1272.626	0.808	0.0155	<0.001
	3-solution	1229.203	1266.753	1222.517	0.889	0.0373	<0.001
	4-solution	1212.648	1260.927	1204.052	0.884	0.6893	<0.001
	5-solution	1194.023	1253.03	1183.516	0.871	0.3633	<0.001

*Abbreviations*: *AIC* the Akaike information criterion, *BIC* the Bayesian information criterion (BIC), *aBIC* the adjusted Bayesian information criterion (aBIC), *A-LRT* the adjusted Vuong-Lo-Mendell-Rubin likelihood ratio test, *BLRT* Bootstrapped Likelihood Ratio Test (BLRT)

**Fig. 2:**
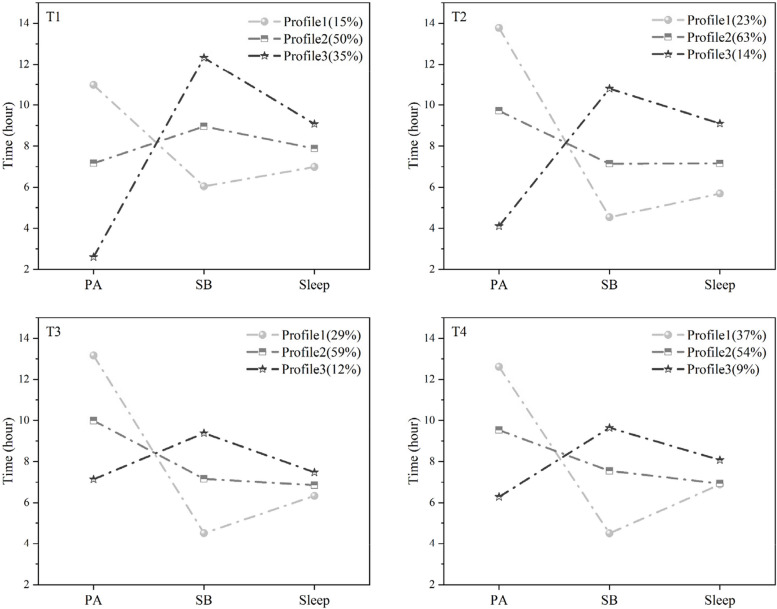
Latent profile of 24-hour movement behaviors. PA: Physical Activity; SB: Sedentary Behavior

Mean values of PA, SB and sleep for each profile at each time point are presented in Figure 2. After the profile-based examination of the characteristics of movement behavior patterns, profiles 1, 2, and 3 were named “Active, Non-sedentary, and Short sleep”, “Active and Sedentary”, and “Inactive and Sedentary”, respectively. Participants in Profile 1 showed a higher level of PA than those in other profiles, and SB was lower than the other subgroups at all four points of time, at about 4-6 hours. In addition, participants in Profile 1 slept less than those in other profiles (below 7 hours). Therefore, the 1-profile model was named “Active, Non-sedentary, and Short Sleep”. Given that the participants in Profile 2 and Profile 3 had a sleep duration of approximately 7-9 hours at the four points of time, their sleep duration falls within the range considered normal [46]. Therefore, the naming of subgroups was mainly based on PA and SB. Profile 2 was named “Active and Sedentary”, which was defined by a mean physical activity time greater than 7 hours and sedentary time greater than 7 hours. Profile 3 was characterized by a lower duration of PA (2-7 hours) and a higher duration of SB (over 9 hours) than the other subgroups at all four points of time; therefore, it was named “Inactive and Sedentary”.

**Fig. 3:**
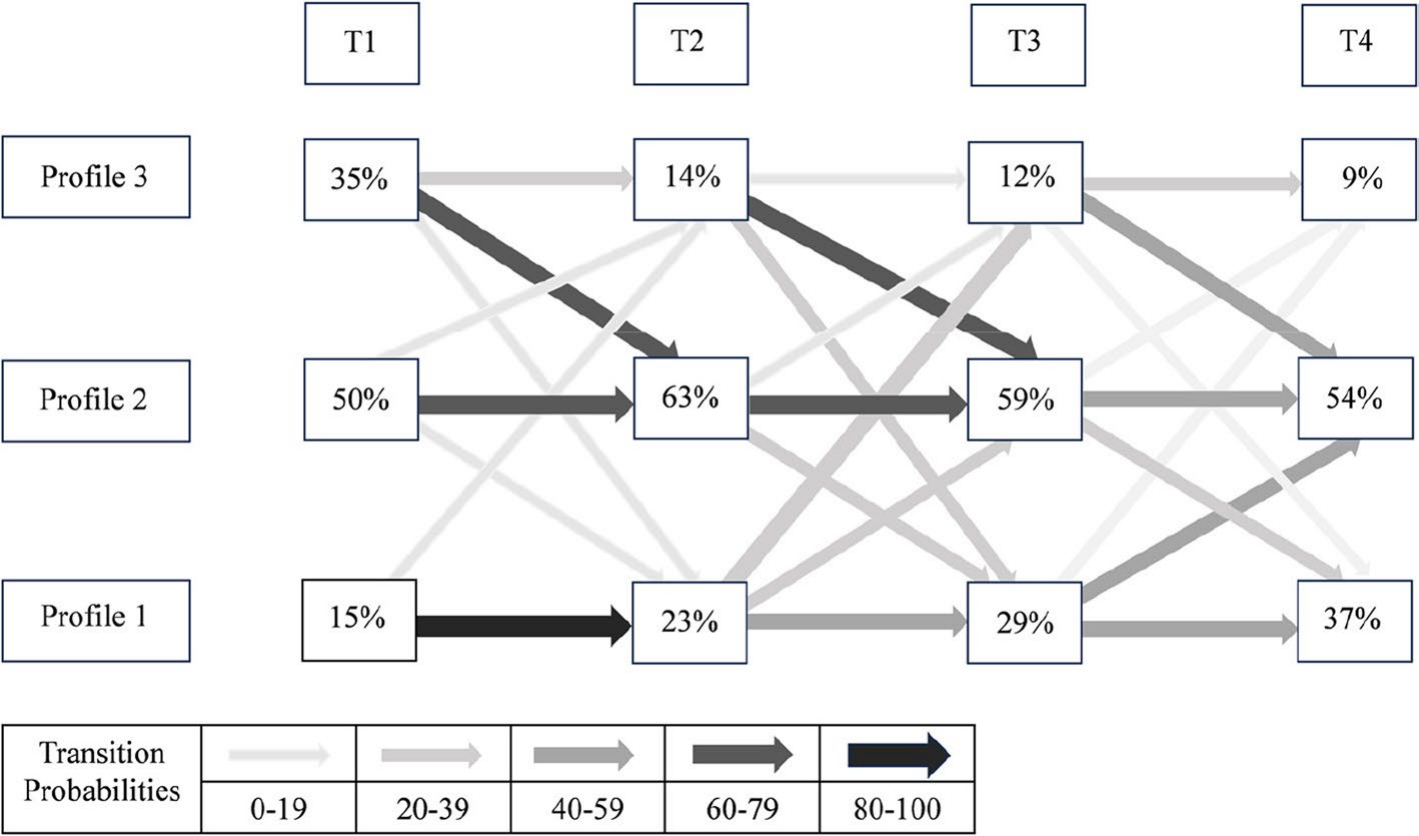
The percentage in each of the three profiles and transition probabilities from T1 to T2, T2 to T3, and T3 to T4. Profile 1: “Active, Non-sedentary and Short sleep”; Profile 2: “Active and Sedentary”; Profile 3: “Inactive and Sedentary”

### Transitions between 24-hour movement behavior profiles in people after stroke

We next assessed the stability of the 24-hour movement behavior profiles at four time points by conducting LTA. Table 4 shows the transitional probabilities of movement behavior from T1 to T2, T2 to T3, and T3 to T4. The diagonal of the transition matrix represents the probability that a participant will remain in the original profile. The proportions and transitional patterns of people after stroke in each profile at T1, T2, T3, and T4 are shown in Fig. 3. Participants in the “Active, Non-sedentary, and Short Sleep” profile were highly likely to stay in the original latent profile, especially within one month (T1→T2, 85.9%). The probability of participants in the “Active and Sedentary” profile staying in the original profile was also high, although the probabilities decreased over time, with T1→T2 (74.5%), T2→T3 (66.3%), and T3→T4 (56.3%). However, those in the “Inactive and Sedentary” profile exhibited a higher probability of transitioning to “Active and Sedentary” after discharge.

**Table 4:** Transition probabilities for the 24-hour movement behavior patterns

	Profile 1	Profile 2	Profile 3
T1→T2			
Profile 1	**0.859**	0.000	0.141
Profile 2	0.180	**0.745**	0.075
Profile 3	0.097	0.652	**0.252**
T2→T3			
Profile 1	**0.429**	0.290	0.281
Profile 2	0.247	**0.663**	0.090
Profile 3	0.231	0.763	**0.005**
T3→T4			
Profile 1	**0.524**	0.441	0.035
Profile 2	0.365	**0.563**	0.072
Profile 3	0.105	0.517	**0.378**
T1→T4			
Profile1	**0.531**	0.417	0.052
Profile2	0.265	**0.666**	0.069
Profile3	0.359	0.542	**0.099**

Profile 1: “Active, Non-sedentary and Short Sleep”; Profile 2: “Active and Sedentary”; Profile 3: “Inactive and Sedentary”

In addition, we investigated the stability of the 24-hour movement behavior profiles from T1 to T4 since there may be a greater magnitude of change in 24-hour movement behaviors over this period. The transitional probabilities of 24-hour movement behavior patterns from T1 to T4 are shown in Table 4. The results were similar from T1 to T2, T2 to T3, and T3 to T4. From T1 to T4, participants in the “Active, Non-sedentary and Short sleep” and “Active and Sedentary” profile were highly likely to stay in the original latent profile. However, from T1 to T4, participants in the “Inactive and Sedentary” profile were less likely to stay in the original latentprofile.

### Socio-demographic characteristics of 24-hour movement behavior profiles and transition status

Socio-demographic characteristics of each profile at four time points are described in Supplementary file table S1. There was a statistically significant difference in the place of residence and residential status among the profiles at T2. In terms of the place of residence, profile 1 had the highest proportion of rural (28%) and county seat (8%) and the lowest proportion of urban (64%). Conversely, profile 2 had the lowest proportion of rural participants (5.9%) and the highest proportion of urban participants (89.7%), *p*=0.022. While most people after stroke lived with family across all profiles, profile 1 had the highest proportion of participants who lived alone (16%). In addition, all participants in profile 3 lived with their families (100%), *p*=0.021. There was a statistically significant difference in the age group among the profiles at T3. Profile 1 had the highest proportion of people after stroke who were ≤ 65 years (77.4%) and the lowest proportion of people who were ＞65 years (22.6%). Profile 2 had the highest proportion of participants who were ＞65 years (53.1%) and the lowest proportion of participants who were ≤ 65 years (46.9%). No significant differences were observed between profiles of 24-hour movement behavior in terms of other socio-demographic characteristics at four time points (*p*＞0.05). Socio-demographic characteristics of participants in different transition statuses, including stable, favorable transition and unfavorable transition are described in Supplementary file table S2. A significant difference in educational attainment was observed among different transition statuses from T2 to T3 (*p *= 0.023). Participants who belonged to unfavorable transition had the highest proportion of high school/secondary school (70%). In comparison, people after stroke who belonged to favorable transition had the highest proportion of post-secondary (22.6%) and college and above (22.6%). There were no significant differences in other socio-demographic characteristics among different transition statuses (*p*＞0.05).

### Relationship of transitions in 24-hour movement behavior patterns with physical function and HRQoL

The latent statusof“Active, Non-sedentary and Short Sleep”at T1 and the latent status of“Inactive and Sedentary” at T2 and T3 were removed from the analysis because they contained a very small number of participants. As shown in Tables 5 and 6, when the participants who remained in the same profile over time were selected as the reference group, the different transition patterns were associated with physical function and HRQoL. In terms of physical function, participants who shifted from “Inactive and Sedentary” to “Active and Sedentary” and “Active, Non-sedentary, and Short Sleep” from T1 to T2 had better physical function (β=-0.824, *p*＜0.001; β=-0.779, *p*=0.032)participants who shifted from “Active, Non-sedentary and Short Sleep”to “Inactive and Sedentary”from T2 to T3 had worse physical function (β=0.894,* p*=0.004); participants who shifted from “Inactive and Sedentary” to “Active, Non-sedentary, and Short Sleep” from T1 to T4 had better physical function (β=-0.936,* p*=0.028). In terms of HRQoL, participants who shifted from “Inactive and Sedentary” to “Active and sedentary”from T1 to T2had better HRQoL (β=0.138,* p*=0.01); participants who shifted from “Active and Sedentary” to “Inactive and Sedentary” from T3 to T4 and T1 to T4 had worse HRQoL (β=-0.077,* p*=0.017; β=-0.121,* p*=0.001). Participants who shifted from “Inactive and Sedentary” to “Active, Non-sedentary and Short Sleep” from T1 to T4 had better HRQoL (β=0.069,* p*=0.04).

**Table 5:** Relationship of transitions in 24-hour movement behavior patterns with physical function

Outcome	Time		Profile 1		Profile 2		Profile 3	
			β	*P*	β	*P*	β	*P*
Physical Function (mRS)	T1 to T2	Profile 1	/	/	/	/	/	/
		Profile 2	−0.106	0.555	REF		−0.366	0.222
		Profile 3	−0.779	0.032^*^	−0.824	<0.001^***^	REF	
	T2 to T3	Profile 1	REF		0.452	0.16	0.894	0.004^**^
		Profile 2	0.18	0.24	REF		0.459	0.059
		Profile 3	/	/	/	/	/	/
	T3 to T4	Profile 1	REF		0.474	0.135	0.992	0.238
		Profile 2	−0.316	0.159	REF		0.871	0.052
		Profile 3	/	/	/	/	REF	
	T1 to T4	Profile 1	/	/	/	/	/	/
		Profile 2	−0.243	0.32	REF		0.987	0.06
		Profile 3	−0.936	0.028^*^	−0.744	0.056	REF	

*P < 0.05; **P < 0.01; ***P < 0.001; Profile 1: “Active, Non-sedentary and Short sleep”; Profile 2: “Active and Sedentary”; Profile 3: “Inactive and Sedentary”

**Table 6:** Relationship of transitions in 24-hour movement behavior patterns with HRQoL

Outcome	Time		Profile 1		Profile 2		Profile 3	
			β	*P*	β	*P*	β	*P*
Health-Related Quality of Life	T1 to T2	Profile 1	/	/	/	/	/	/
		Profile 2	0.027	0.479	REF		−0.004	0.946
		Profile 3	0.149	0.105	**0.138**	0.01^*^	REF	
	T2 to T3	Profile 1	REF		−0.061	0.166	−0.043	0.307
		Profile 2	0.032	0.171	REF		−0.055	0.141
		Profile 3	/	/	/	/	/	/
	T3 to T4	Profile 1	REF		−0.031	0.217	−0.024	0.716
		Profile 2	0.031	0.056	REF		**−0.077**	0.017^*^
		Profile 3	/	/	/	/	/	/
	T1 to T4	Profile 1	/	/	/	/	/	/
		Profile 2	0.021	0.23	REF		**−0.121**	0.001^**^
		Profile 3	**0.069**	0.04^*^	0.049	0.116	REF	

*P < 0.05; **P < 0.01; ***P < 0.001; Profile 1: “Active, Non-sedentary and Short sleep”; Profile 2: “Active and Sedentary”; Profile 3: “Inactive and Sedentary”; HRQoL: Health-Related Quality of Life

## Discussion

This study employed an individual-centered approach to examine the heterogeneity, similarities, and variations of 24-hour movement behaviors in people after stroke during rehabilitation using objective 24-hour movement behavior data collected by wristband smartwatches. Three distinct profiles of 24-hour movement behaviors were identified, and transitions between profiles of 24-hour movement behaviors over time were associated with HRQoL and physical function. To the best of our knowledge, this is the first study of 24-hour movement behavior patterns in people after stroke. This study identified three patterns of 24-hour movement behavior among people after stroke: “Active, Non-sedentary, and Short Sleep,” “Active and Sedentary,” and “Inactive and Sedentary.” Over time, there was an increase in the number of participants who were classified as “Active, Non-sedentary, and Short Sleep,” while the percentage of participants categorized as “Inactive and Sedentary” decreased.This shows that people after stroke tend to improve their movement behavior patterns over time during the six months after discharge, which is consistent with the findings of van der Laag et al. [22]. Our study also found that people after stroke who are “Active and Sedentary” accounted for more than half of the sample at each of the four points of time. Our study supports the finding of Fini et al [7], who reported that the PA duration of people after stroke was higher in the chronic phase than in the subacute phase; however, they exhibited a long sedentary time, regardless of the disease phase. The majority of our participants engaged in adequate PA, yet prolonged sedentary behavior was still observed. Prolonged SB was independently associated with the increased risk of cardiometabolic problems [47]; therefore, future studies are recommended to prioritize and apply interventions aimed at reducing sedentary time among people suffering from stroke. Transition analyses at each stage demonstrated that the participants who were in the “Active, Non-sedentary, and Short Sleep” profile had very high stability of movement behavior patterns over the month. However, from one to three months and three to six months, there was a 57.1% and 47.6% likelihood of shifting to a more unfavorable movement behavior pattern during wakefulness. Interventions for this subgroup need to focus on maintaining the levels of PA and SB, especially within the first month after discharge. In contrast, the stability of movement behavior patterns among participants categorized as “Active and Sedentary” remained high over a six-month period, whereas those in the "Inactive and Sedentary" group exhibited lower stability and a greater tendency to transition into the “Active and Sedentary”. This can be attributed to the gradual recovery of physical functions in people after stroke after discharge as they continue to recover from the disease, allowing previously inactive individuals to gradually increase their PA. However, they were highly likely to remain sedentary, possibly due to the low knowledge of people after stroke about the negative effects and health risks of SB [48]. To reduce the risk of cardiovascular diseases in people with stroke who have a sedentary lifestyle, it is important to promptly and effectively decrease their sedentary time. However, no evidence of effective interventions has been found on SB among people after stroke [49]. More high-quality studies are needed to help people after stroke become less sedentary in the future. PA can be categorized as light physical activity (LPA) and moderate-to-vigorous physical activity (MVPA) [50]. MVPA is essential for improving the health of people after stroke, but stroke-induced disability may limit participation in MVPA for people after stroke. LPA is more accessible than MVPA and more likely to be part of daily life. Consequently, it may be easier to encourage people after stroke to break SB with LPA. We found statistically significant differences in the sociodemographic characteristics among the latent profiles and transition patterns. When comparing profile 2 or 3 to profile 1, there was a higher proportion of old people (＞65 years), urban, and living with families. Our findings are consistent with the findings of previous studies where older age was associated with long sedentary time and physical inactivity [6, 51]. However, the relationship between residential status and movement behavior in this study was inconsistent with that observed in previous studies [51]. This may be due to different socio-cultural backgrounds. In addition, when comparing favorable transitions to unfavorable transitions, there was a higher proportion of education equivalent or more than post-secondary. This highlights the importance of education in identifying different transition patterns. Educational intervention may be an effective measure to promote the shift to favorable movement behavior patterns in people after stroke.

Transitions in 24-hour movement behavior patterns 6 months after discharge were significantly associated with changes in physical function. This suggests that increasing PA and decreasing SB in the early stages of rehabilitation can significantly improve functional outcomes in people after stroke, which is consistent with the findings of previous studies [12, 13, 52]. Healthcare professionals should focus on the interrelationship between 24-hour movement behaviors and how they change over time. However, the relationship between transitions in 24-hour movement behavior patterns and physical function was inconsistent in this study. The study results demonstrated a relationship between improvement in movement behavior during wakefulness and a decline in physical functioning three months after discharge, although the relationship was not statistically significant. To explain this result, it can be stated that PA intensity has been associated with functional recovery [53], and the guidelines recommend adequate MVPA for people after stroke [3]. However, this study did not refine PA into LPA and MVPA, which may have influenced the results. Alternatively, the effects of movement patterns on functional outcomes may decrease as people after stroke mostly regain their physical abilities within six months after discharge.

Previous studies indicated that exercise initiated within six months after discharge can moderately improve the HRQoL of people after stroke [10], consistent with our findings. Specifically, HRQoL was higher among people after stroke who shifted from “Inactive and Sedentary” to “Active and Sedentary”. People after stroke who shifted from “Active and Sedentary” to “Inactive and Sedentary” had lower levels of HRQoL. However, neither people after stroke who shifted to “Active, Non-sedentary and Short sleep” profile nor those who shifted from “Active, Non-sedentary and Short sleep” profile to other profiles did not have a significantly higher or lower level of HRQoL. This finding should be interpreted cautiously. Engagement in regular exercise can increase strength, endurance, and cardiorespiratory fitness, all of which can improve HRQoL [10, 54]. However, motor deficits explain only 17.5-24.1% of the EQ-5D variance [55]. In addition to mobility, quality of life encompasses multiple aspects, such as pain and anxiety. People after stroke in “Active, Non-sedentary and Short sleep” profile follow a better movement behavior pattern when awake, but their sleep duration is short. Short sleep (<7h) is associated with increased depression, fatigue, and anxiety [56], which can negatively affect HRQoL [57, 58]. Nevertheless, future studies are recommended to further explore the effects of transitions between profiles of 24-hour movement behavior on people after stroke. Future updates to the exercise guidelines for people after stroke should also specify how patients allocate their time to sleep, PA, and SB to achieve optimal health benefits.

### Limitations and prospects

Due to device limitations, this study did not differentiate between MVPA and LPA, as well as the purpose of sedentary time, resulting in incomplete information regarding potential profiles. The smartwatch we used could not differentiate between standing and PA, potentially overestimating PA. To address this limitation, future studies should utilize more precise equipment or integrate equipment with questionnaires to furnish more detailed and accurate insights into PA and SB. Secondly, the limitations of this study should also be considered when interpreting the results. Since the study sample represented only people after stroke admitted to a single hospital in China, the study findings should be cautiously generalized to other populations. As a result, more studies with larger and more diverse samples are needed to explore the 24-hour movement behavior patterns of people after stroke and to discover more potential patterns. In addition, future studies should further explore the antecedents and consequences of shifting movement behavior patterns in people after stroke.

## Conclusions

The 24-hour movement behavior of people after stroke in this study was diverse and showed varying levels of stability over time. Later physical function and HRQoL were also correlated with the transitions between profiles of 24-hour movement behaviors. This study provides information to help individuals with unhealthy 24-hour movement behavior patterns receive more targeted interventions. The study findings also highlighted the importance of promptly identifying and addressing sedentary issues, which exhibit a significant level of consistency over time.

## Supplementary Information

Supplementary Material 1.

Supplementary Material 2.

## Data Availability

The datasets used and/or analysed during the current study are available from the corresponding author on reasonable request.

## Abbreviations

LPA: Latent profile analysis
LTA: Latent transition analysis
PA: Physical Activity
LPA: Light Physical Activity
MVPA: Moderate-to-Vigorous Physical Activity
SB: Sedentary Behavior
ADL: Activities of Daily Living
BI: Barthel Index
NIHSS: National Institute of Health Stroke Scale
EQ-5D-5L: EuroQol Five-Dimensions Five-Level Questionnaire
mRS: modified Rankin Scale
HRQoL: Health-Related Quality of Life
BMI: body mass index
UEBMI: urban employee basic medical insurance
URRBMI: urban and rural resident basic medical insurance
AIC: Akaike Information Criterion
BIC: Bayesian Information Criterion
ABIC: Adjusted Bayesian Information Criterion
A-LRT: Adjusted Vuong-Lo-Mendell-Rubin likelihood ratio test
BLRT: Bootstrap Likelihood Ratio Test
