# Supplementary material for: Longitudinal relationship between 24-Hour Movement behavior patterns and physical function and quality of life after stroke: a latent transition analysis
Source: Int J Behav Nutr Phys Act. 2024 Dec 18;21:141. doi: 10.1186/s12966-024-01689-1 (PMC11656942; doi:10.1186/s12966-024-01689-1)
Supplement: Supplementary file 2 — Supplementary Material 2. [file 12966_2024_1689_MOESM2_ESM.docx]

Supplemental table 1. Socio-demographic characteristics of each profile of 24-hour movement behavior at T1, T2, T3 and T4 n (%).

| Demographics | T1 | | | | |  | T2 | | | | |  | T3 | | | | |  | T4 | | | | |
| --- | --- | --- | --- | --- | --- | --- | --- | --- | --- | --- | --- | --- | --- | --- | --- | --- | --- | --- | --- | --- | --- | --- | --- |
|  | Profile1 | Profile2 | Profie3 | 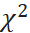 | *P* |  | Profile1 | Profile2 | Profie3 | 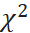 | *P* |  | Profile1 | Profile2 | Profie3 | 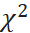 | *P* |  | Profile1 | Profile2 | Profie3 | 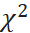 | *P* |
|  | (n=16) | (n=54) | (n=38） |  |  |  | (n=25) | (n=68) | (n=15) |  |  |  | (n=31) | (n=64) | (n=13) |  |  |  | (n=40) | (n=58) | (n=10) |  |  |
| Age (years) |  |  |  | 2.156^a^ | 0.34 |  |  |  |  | 4.226^a^ | 0.121 |  |  |  |  | 7.969^a^ | **0.019** |  |  |  |  | 4.074^a^ | 0.130 |
| ≤65 | 11(68.8) | 27(50) | 23(60.5) |  |  |  | 18(72) | 37(54.4) | 6(40) |  |  |  | 24(77.4) | 30(46.9) | 7(53.8) |  |  |  | 26(65) | 32(55.2) | 3(30) |  |  |
| > 65 | 5(31.3) | 27(50) | 15(39.5) |  |  |  | 7(28) | 31(45.6) | 9(60) |  |  |  | 7(22.6) | 34(53.1) | 6(46.2) |  |  |  | 14(35) | 26(44.8) | 7(70) |  |  |
| Gender |  |  |  | 1.325^a^ | 0.516 |  |  |  |  | 1.237^a^ | 0.539 |  |  |  |  | 1.894^a^ | 0.388 |  |  |  |  | 0.788^a^ | 0.674 |
| Male | 9(56.3) | 31(57.4) | 26(68.4) |  |  |  | 14(56) | 41(60.3) | 11(73.3) |  |  |  | 18(58.1) | 42(65.6) | 6(46.2) |  |  |  | 26(65) | 35(60.3) | 5(50) |  |  |
| Female | 7(43.8) | 23(42.6) | 12(31.6) |  |  |  | 11(44) | 27(39.7) | 4(26.7) |  |  |  | 13(41.9) | 22(34.4) | 7(53.8) |  |  |  | 14(35) | 23(39.7) | 5(50) |  |  |
| BMI (kg/m2) |  |  |  | 4.701^b^ | 0.285 |  |  |  |  |  |  |  |  |  |  | 4.062^b^ | 0.337 |  |  |  |  | 1.736^b^ | 0.802 |
| <18.5 | 1(6.3) | 0(0) | 2(5.3) |  |  |  | 1(4) | 2(2.9) | 0(0) |  |  |  | 2(6.5) | 1(1.6) | 0(0) |  |  |  | 2(5) | 1(1.7) | 0(0) |  |  |
| 18-25 | 7(43.8) | 27(50) | 14(36.8) |  |  |  | 12(48) | 31(45.6) | 5(33.3) |  |  |  | 10(32.3) | 31(48.4) | 7(53.8) |  |  |  | 19(47.5) | 25(43.1) | 4(40) |  |  |
| ≥25 | 8(50) | 27(50) | 22(57.9) |  |  |  | 12(48) | 35(51.5) | 10(66.7) |  |  |  | 19(61.3) | 32(50) | 6(46.2) |  |  |  | 19(47.5) | 32(55.2) | 6(60) |  |  |
| Occupation |  |  |  | 2.150^a^ | 0.341 |  |  |  |  | 1.114^a^ | 0.573 |  |  |  |  | 5.178^a^ | 0.075 |  |  |  |  | 3.215^a^ | 0.200 |
| Employed | 7(43.8) | 21(38.9) | 10(26.3) |  |  |  | 11(44) | 22(32.4) | 5(33.3) |  |  |  | 16(51.6) | 18(28.1) | 4(30.8) |  |  |  | 16(40) | 21(36.2) | 1(10) |  |  |
| Retirement/ Unemployed | 9(56.3) | 33(61.1) | 28(73.7) |  |  |  | 14(56) | 46(67.6) | 10(66.7) |  |  |  | 15(48.4) | 46(71.9) | 9(69.2) |  |  |  | 24(60) | 37(63.8) | 9(90) |  |  |
| Smoking history |  |  |  | 1.514a | 0.469 |  |  |  |  | 0.239^a^ | 0.887 |  |  |  |  | 0.262^a^ | 0.877 |  |  |  |  | 0.887^a^ | 0.642 |
| Smoker | 5(31.3) | 18(33.3) | 17(44.7) |  |  |  | 10(40) | 24(35.3) | 6(40) |  |  |  | 12(38.7) | 24(37.5) | 4(30.8) |  |  |  | 17(42.5) | 20(34.5) | 3(30) |  |  |
| Non-smoker | 11(68.8) | 36(66.7) | 21(55.3) |  |  |  | 15(60) | 44(64.7) | 9(60) |  |  |  | 19(61.3) | 40(62.5) | 9(69.2) |  |  |  | 23(57.5) | 38(65.5) | 7(70) |  |  |
| Drinking history |  |  |  | 3.474a | 0.176 |  |  |  |  | 0.438^a^ | 0.803 |  |  |  |  | 2.401^a^ | 0.301 |  |  |  |  | 1.272^a^ | 0.529 |
| Drinker | 4(25) | 15(27.8) | 17(44.7) |  |  |  | 8(32) | 24(35.3) | 4(26.7) |  |  |  | 10(32.3) | 24(37.5) | 2(15.4) |  |  |  | 16(40) | 17(29.3) | 3(30) |  |  |
| Non-drinker | 12(75) | 39(72.2) | 21(55.3) |  |  |  | 17(68) | 44(64.7) | 11(73.3) |  |  |  | 21(67.7) | 40(62.5) | 11(84.6) |  |  |  | 24(60) | 41(70.7) | 7(30) |  |  |
| Education |  |  |  | 3.757b | 0.724 |  |  |  |  | 3.589^b^ | 0.748 |  |  |  |  | 7.877^b^ | 0.224 |  |  |  |  | 6.764^b^ | 0.317 |
| Elementary school and below | 5(31.3) | 15(27.8) | 9(23.7) |  |  |  | 6(24) | 20(29.4) | 3(20) |  |  |  | 6(19.4) | 20(31.3) | 3(23.1) |  |  |  | 15(37.5) | 12(20.7) | 2(20) |  |  |
| High school/secondary school | 5(31.3) | 27(50) | 16(42.1) |  |  |  | 12(48) | 31(45.6) | 5(33.3) |  |  |  | 12(38.7) | 27(42.2) | 9(69.2) |  |  |  | 12(30) | 30(51.7) | 6(60） |  |  |
| Post-secondary | 4(25) | 7(13) | 9(23.7) |  |  |  | 5(20) | 11(16.2) | 4(26.7) |  |  |  | 8(25.8) | 12(18.8) | 0(0) |  |  |  | 8(20) | 10(17.2) | 2(20) |  |  |
| College and above | 2(12.5) | 5(9.3) | 4(10.5) |  |  |  | 2(8) | 6(8.8) | 3(20) |  |  |  | 5(16.1) | 5(7.8) | 1(7.7) |  |  |  | 5(12.5) | 6(10.3) | 0 |  |  |
| Marital status |  |  |  | 4.282b | 0.115 |  |  |  |  | 0.781^b^ | 0.718 |  |  |  |  | 0.781^b^ | 0.728 |  |  |  |  | 4.078^b^ | 0.113 |
| Married | 12(75) | 49(90.7) | 36(94.7) |  |  |  | 22(88) | 62(91.2) | 13(86.7) |  |  |  | 28(90.3) | 58(90.6) | 11(84.6) |  |  |  | 34(85) | 55(94.8) | 8(80) |  |  |
| Unmarried/Divorced /Widowed | 4(25) | 5(9.3) | 2(5.3) |  |  |  | 3(12) | 6(8.8) | 2(13.3) |  |  |  | 3(9.7) | 6(9.4) | 2(15.4) |  |  |  | 6(15) | 3(5.2) | 2(20) |  |  |
| Place of residence |  |  |  | 5.160b | 0.225 |  |  |  |  | 9.975^b^ | **0.022** |  |  |  |  | 2.662^b^ | 0.630 |  |  |  |  | 1.611^b^ | 0.837 |
| Rural | 3(18.8) | 6(11.1) | 5(13.2) |  |  |  | 7(28） | 4(5.9) | 3(20) |  |  |  | 2(6.5) | 10(15.6) | 2(15.4) |  |  |  | 4(10) | 9(15.5) | 1(10) |  |  |
| County seat | 2(12.5) | 3(5.6) | 0(0) |  |  |  | 2(8) | 3(4.4) | 0 |  |  |  | 1(3.2) | 3(4.7) | 1(7.7) |  |  |  | 3(7.5) | 2(3.4) | 0 |  |  |
| Urban | 11(68.8) | 45(83.3) | 33(86.8) |  |  |  | 16(64) | 61(89.7) | 12(80) |  |  |  | 28(90.3) | 51(79.7) | 10(76.9) |  |  |  | 33(82.5) | 47(81) | 9(90) |  |  |
| Residential status |  |  |  | 2.524b | 0.282 |  |  |  |  | 6.829^b^ | **0.021** |  |  |  |  | 1.516^b^ | 0.499 |  |  |  |  | 0.260^b^ | 1.000 |
| Live alone | 2(12.5) | 2(3.7) | 1(2.6) |  |  |  | 4(16) | 1(1.5) | 0 |  |  |  | 2(6.5) | 2(3.1) | 1(7.7) |  |  |  | 2(5) | 3(5.2) | 0 |  |  |
| Live with families | 14(87.5) | 52(96.3) | 37(97.4) |  |  |  | 21(84) | 67(98.5) | 15(100) |  |  |  | 29(93.5) | 62(96.9) | 12(92.3) |  |  |  | 38(95) | 55(94.8) | 10(100) |  |  |
| Family per capita monthly income(yuan) |  |  |  | 2.967b | 0.578 |  |  |  |  | 6.337^b^ | 0.151 |  |  |  |  | 2.330^b^ | 0.673 |  |  |  |  | 4.623^b^ | 0.306 |
| <3000 | 3(18.8) | 4(7.4) | 2(5.3) |  |  |  | 5(20) | 4(5.9) | 0 |  |  |  | 2(6.5) | 6(9.4) | 1(7.7) |  |  |  | 3(7.5) | 6(10.3) | 0 |  |  |
| 3000~5000 | 9(56.3) | 38(70.4) | 27(71.1) |  |  |  | 14(56) | 47(69.1) | 13(86.7) |  |  |  | 19(61.3) | 46(71.9) | 9(69.2) |  |  |  | 24(60) | 43(74.1) | 7(70) |  |  |
| >5000 | 4(25) | 12(22.2) | 9(23.7) |  |  |  | 6(24) | 17(25) | 2(13.3) |  |  |  | 10(32.3) | 12(18.8) | 3(23.1) |  |  |  | 13(32.5) | 9(15.5) | 3(30) |  |  |
| Medical insurance |  |  |  | 1.211b | 0.934 |  |  |  |  | 2.388^b^ | 0.672 |  |  |  |  | 1.140^b^ | 1 |  |  |  |  | 1.231^b^ | 0.957 |
| UEBMI | 2(12.5) | 5(9.3) | 3(7.9) |  |  |  | 2(8) | 8(11.8) | 0 |  |  |  | 3(9.7) | 6(9.4) | 1(7.7) |  |  |  | 3(7.5) | 6(10.3) | 1(10) |  |  |
| URRBMI | 14(87.5) | 48(88.9) | 34(89.5) |  |  |  | 23(92) | 58(85.3) | 15(100) |  |  |  | 28(90.3) | 56(87.5) | 12(92.3) |  |  |  | 36(90) | 51(87.9) | 9(90) |  |  |
| Self-financed | 0(0) | 1(1.9) | 1(2.6) |  |  |  | 0 | 2(2.9) | 0 |  |  |  | 0 | 2(3.1) | 0 |  |  |  | 1(2.5) | 1(1.7) | 0 |  |  |

**Notes:** ^a^: chi-square tests，^b^: Fisher’s exact test

BMI, Body Mass Index, calculated as weight in kilograms divided by height in meters squared; UEBMI, Urban Employee Basic Medical Insurance; URRBMI, Urban and Rural Resident Basic Medical Insurance

Supplemental table 2. Socio-demographic characteristics of transition status n (%).

| Demographics | T1 to T2 | | | |  | T2 to T3 | | | | | | | |  | | T3 to T4 | | | | | | | |  | | T1 to T4 | | | | | | | |  |
| --- | --- | --- | --- | --- | --- | --- | --- | --- | --- | --- | --- | --- | --- | --- | --- | --- | --- | --- | --- | --- | --- | --- | --- | --- | --- | --- | --- | --- | --- | --- | --- | --- | --- | --- |
|  | Stable | Unfavorable transition | Favorable transition | 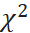/*P* |  | | Stable | | Unfavorable transition | | Favorable transition | | 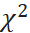/*P* | |  | | Stable | | Unfavorable transition | | Favorable transition | | 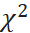/*P* | |  | | Stable | | Unfavorable transition | | Favorable transition | | 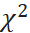/*P* | |
|  | (n=64) | (n=6) | (n=38) |  |  | | (n=57) | | (n=20) | | (n=31) | |  |  |  | | (n=58) | | (n=19) | | (n=31) | |  |  |  | | (n=44) | | (n=11) | | (n=53) | |  |  |
| Age (years) |  |  |  | 3.935^b^/0.153 |  | |  | |  | |  | | 0.736^a^/0.692 | |  | |  | |  | |  | | 2.385^a^/0.303 | |  | |  | |  | |  | | 2.657^a^/0.265 | |
| ≤65 | 37 (57.8) | 1 (16.7) | 23 (60.5) |  |  | | 30 (52.6) | | 12 (60) | | 19 (61.3) | |  | |  | | 29 (50) | | 13 (68.4) | | 19 (61.3) | |  | |  | | 21 (47.7) | | 6 (54.5) | | 34 (64.2) | |  | |
| > 65 | 27(42.2) | 5(83.3) | 15 (39.5) |  |  | | 27(47.4) | | 8 (40) | | 12 (38.7) | |  | |  | | 29 (50) | | 6 (31.6) | | 12 (38.7) | |  | |  | | 23 (52.3) | | 5 (45.5) | | 19 (35.8) | |  | |
| Gender |  |  |  | 0.268^b^/0.946 |  | |  | |  | |  | | 0.832^a^/0.660 | |  | |  | |  | |  | | 0.248^a^/0.883 | |  | |  | |  | |  | | 1.751^a^/0.417 | |
| Male | 38 (59.4) | 4 (66.7) | 24 (63.2) |  |  | | 33 (57.9) | | 12 (60) | | 21 (67.7) | |  | |  | | 35 (60.3) | | 11(57.9) | | 20 (64.5) | |  | |  | | 26 (59.1) | | 5 (45.5) | | 35 (66) | |  | |
| Female | 26 (40.6) | 2(33.3) | 14 (36.8) |  |  | | 24 (42.1) | | 8 (40) | | 10 (32.3) | |  | |  | | 23 (39.7) | | 8 (42.1) | | 11 (35.5) | |  | |  | | 18 (40.9) | | 6 (54.5) | | 18 (34) | |  | |
| BMI (kg/m2) |  |  |  | 2.300^b^/0.700 |  | |  | |  | |  | | 2.342^b^/0.659 | |  | |  | |  | |  | | 6.322^b^/0.132 | |  | |  | |  | |  | | 3.382b/0.495 | |
| <18.5 | 1 (1.6) | 0 (0) | 2 (5.2) |  |  | 2 (3.5) | | 0 (0) | | 1 (3.2) | |  | |  | | 1 (1.7) | | 1 (5.3) | | 1 (3.2) | |  | |  | | 0(0) | | 1 (9.1) | | 2 (3.8) | |  | |  |
| 18.5-25 | 27 (42.2) | 3 (50) | 18 (47.4) |  |  | 26 (45.6) | | 11 (55) | | 11 (35.5) | |  | |  | | 28 (48.3) | | 4 (21.1) | | 16 (51.6) | |  | |  | | 20(45.5) | | 5 (45.5) | | 23 (43.4) | |  | |  |
| ≥25 | 36 (56.3) | 3 (50) | 18 (47.4) |  |  | 29 (50.9) | | 9 (45) | | 19 (61.3) | |  | |  | | 29 (50) | | 14 (73.7) | | 14 (45.2) | |  | |  | | 24(54.5) | | 5 (45.5) | | 28 (52.8) | |  | |  |
| Occupation |  |  |  | 1.123^b^/0.565 |  |  | |  | |  | | 1.541^a^/0.463 | |  | |  | |  | |  | | 0.729^a^/0.694 | |  | |  | |  | |  | | 0.567a/0.753 | |  |
| Employed | 25 (39.1) | 2 (33.3) | 11 (28.9) |  |  | 17 (29.8) | | 8 (40) | | 13 (41.9) | |  | |  | | 22 (37.9) | | 7 (36.8) | | 9 (29) | |  | |  | | 15 (34.1) | | 5 (45.5) | | 18 (34) | |  | |  |
| Retirement/ Unemployed | 39 (60.9) | 4 (66.7) | 27 (71.1) |  |  | 40 (70.2) | | 12 (60) | | 18 (58.1) | |  | |  | | 36 (62.1) | | 12 (63.2) | | 22 (71) | |  | |  | | 29 (65.9) | | 6 (54.5) | | 35 (66) | |  | |  |
| Smoking history |  |  |  | 1.004^b^/0.642 |  |  | |  | |  | | 0.205^a^/0.902 | |  | |  | |  | |  | | 0.300^a^/0.861 | |  | |  | |  | |  | | 2.140^a^/0.343 | |  |
| Smoker | 24 (37.5) | 1 (16.7) | 15 (39.5) |  |  | 20 (35.1) | | 8 (40) | | 12 (38.7) | |  | |  | | 22 (37.9) | | 6 (31.6) | | 12 (38.7) | |  | |  | | 16(36.4) | | 2 (18.2) | | 22 (41.5) | |  | |  |
| Non-smoker | 40 (62.5) | 5 (83.3) | 23 (60.5) |  |  | 37 (64.9) | | 12 (60) | | 19 (61.3) | |  | |  | | 36 (62.1) | | 13 (68.4) | | 19 (61.3) | |  | |  | | 28(63.6) | | 9 (81.8) | | 31 (58.5) | |  | |  |
| Drinking history |  |  |  | 2.249^b^/0.356 |  |  | |  | |  | | 0.674^a^/0.714 | |  | |  | |  | |  | | 0.520^a^/0.771 | |  | |  | |  | |  | | 4.788a/0.091 | |  |
| Drinker | 19 (29.7) | 1 (16.7) | 16 (42.1) |  |  | 21 (36.8) | | 6 (30) | | 9 (29) | |  | |  | | 20 (34.5) | | 5 (26.3) | | 11 (35.5) | |  | |  | | 13 (29.5) | | 1 (9.1) | | 22 (41.5) | |  | |  |
| Non-drinker | 45 (70.3) | 5 (83.3) | 22 (57.9) |  |  | 36 (63.2) | | 14 (70) | | 22 (71) | |  | |  | | 38 (65.5) | | 14 (73.7) | | 20 (64.5) | |  | |  | | 31 (70.5) | | 10 (90.9) | | 31 (58.5) | |  | |  |
| Education |  |  |  | 4.675^b^/0.566 |  |  | |  | |  | | 14.031^b^/**0.023** | | | |  | |  | |  | | 10.271^b^/0.100 | |  | |  | |  | |  | | 5.593^b^/0.456 | |  |
| Elementary school and below | 18 (28.1) | 2 (33.3) | 9 (23.7) |  |  | 20 (35.1) | | 4 (20) | | 5 (16.1) | |  | |  | | 15 (25.9) | | 2 (10.5) | | 12 (38.7) | |  | |  | | 11 (25) | | 2 (18.2) | | 16 (30.2) | |  | |  |
| High school/secondary school | 30 (46.9) | 1 (16.7) | 17 (44.7) |  |  | 22 (38.5) | | 14 (70) | | 12 (38.7) | |  | |  | | 26 (44.8) | | 9 (47.4) | | 13 (41.9) | |  | |  | | 22 (50) | | 7 (63.6) | | 19 (35.8) | |  | |  |
| Post-secondary | 9 (14.1) | 2 (33.3) | 9 (23.7) |  |  | 12 (21.1) | | 1 (5) | | 7 (22.6） | |  | |  | | 8 (13.8) | | 7 (36.8) | | 5 (16.1) | |  | |  | | 9 (20.5) | | 1 (9.1) | | 10 (18.9) | |  | |  |
| College and above | 7 (10.9) | 1 (16.7) | 3 (7.9) |  |  | 3 (5.3) | | 1 (5) | | 7 (22.6） | |  | |  | | 9 (15.5) | | 1 (5.3) | | 1 (3.2) | |  | |  | | 2 (4.5) | | 1 (9.1) | | 8 (15.1) | |  | |  |
| Marital status |  |  |  | 4.129^b^/0.119 |  |  | |  | |  | | 0.872^b^/0.635 | |  | |  | |  | |  | | 2.731^b^/0.266 | |  | |  | |  | |  | | 3.652^b^/0.127 | |  |
| Married | 57 (89.1) | 4 (66.7) | 36 (94.7) |  |  | 52 (91.2) | | 17 (85) | | 28 (90.3) | |  | |  | | 50 (86.2) | | 19 (100) | | 28 (90.3) | |  | |  | | 41 (93.2) | | 8 (72.7) | | 48 (90.6) | |  | |  |
| Unmarried/Divorced /Widowed | 7 (10.9) | 2 (33.3) | 2 (5.3) |  |  | 5 (8.8) | | 3 (15) | | 3 (9.7) | |  | |  | | 8 (13.8) | | 0 (0) | | 3 (9.7) | |  | |  | | 3 (6.8) | | 3 (27.3) | | 5 (9.4) | |  | |  |
| Place of residence |  |  |  | 3.599^b^/0.420 |  |  | |  | |  | | 7.542^b^/0.007 | |  | |  | |  | |  | | 7.068^b^/0.092 | |  | |  | |  | |  | | 1.996^b^0.744 | |  |
| Rural | 9 (14.1) | 0 (0) | 5 (13.2) |  |  | 5 (8.8) | | 6 (30) | | 3 (9.7) | |  | |  | | 11 (19) | | 0 (0) | | 3 (9.7) | |  | |  | | 6 (13.6) | | 2 (18.2) | | 6 (11.3) | |  | |  |
| County seat | 5 (7.8) | 0 (0) | 0 (0) |  |  | 4 (7) | | 1 (5) | | 0 (0) | |  | |  | | 2 (3.4) | | 0 (0) | | 3 (9.7) | |  | |  | | 2 (4.5) | | 1 (9.1) | | 2 (3.8) | |  | |  |
| Urban | 50 (78.1) | 6 (100) | 33 (86.8) |  |  | 48 (84.2) | | 13 (65) | | 28 (90.3) | |  | |  | | 45 (77.6) | | 19 (100) | | 25 (80.6) | |  | |  | | 36 (81.8) | | 8 (72.7) | | 45 (84.9) | |  | |  |
| Residential status |  |  |  | 0.331^b^/1.000 |  |  | |  | |  | | 4.801^b^/0.074 | |  | |  | |  | |  | | 1.082^b^/0.577 | |  | |  | |  | |  | | 4.113^b^/0.158 | |  |
| Live alone | 3 (4.7) | 0 (0) | 2 (5.3) |  |  | 1 (1.8) | | 3 (15) | | 1 (3.2) | |  | |  | | 4(6.9) | | 0 (0) | | 1 (3.2) | |  | |  | | 1 (2.3) | | 2 (18.2) | | 2 (3.8) | |  | |  |
| Live with families | 61 (95.3) | 6 (100) | 36 (94.7) |  |  | 56 (98.2) | | 17 (85) | | 30 (96.8) | |  | |  | | 54(93.1) | | 19 (100) | | 30 (96.8) | |  | |  | | 43 (97.7) | | 9 (81.8) | | 51 (96.2) | |  | |  |
| Family per capita monthly income(yuan) |  |  |  | 2.273^b^/0.705 |  |  | |  | |  | | 5.352^b^/0.239 | |  | |  | |  | |  | | 0.469^b^/1.000 | |  | |  | |  | |  | | 6.213^b^/0.159 | |  |
| <3000 | 5 (7.8) | 0 (0) | 4 (10.5) |  |  | 6 (10.5) | | 0 (0) | | 3 (15) | |  | |  | | 5 (8.6) | | 1 (5.3) | | 3 (9.7) | |  | |  | | 2 (4.5) | | 3 (27.3) | | 4 (7.5) | |  | |  |
| 3000~5000 | 43 (67.2) | 6 (100) | 25 (65.8) |  |  | 37 (64.9) | | 23 (74.2) | | 14 (70) | |  | |  | | 39 (67.2) | | 14 (73.7) | | 21 (67.7) | |  | |  | | 33 (75) | | 7 (63.6) | | 34 (64.2) | |  | |  |
| >5000 | 16 (25) | 0 (0) | 9 (23.7) |  |  | 14 (24.6) | | 8 (25.8) | | 3 (15) | |  | |  | | 14 (24.1) | | 4 (21.1) | | 7 (22.6) | |  | |  | | 9 (20.5) | | 1 (9.1) | | 15 (28.3) | |  | |  |
| Medical insurance |  |  |  | 1.545^b^/0.941 |  |  | |  | |  | | 1.654^b^/0.872 | |  | |  | |  | |  | | 3.484^b^/0.470 | |  | |  | |  | |  | | 2.201^b^/0.794 | |  |
| UEBMI | 7 (10.9) | 0 (0) | 3 (7.9) |  |  | 6 (10.5) | | 2 (10) | | 2 (6.5) | |  | |  | | 8 (13.8) | | 1 (5.3) | | 1 (3.2) | |  | |  | | 4 (9.1) | | 2 (18.2) | | 4 (7.5) | |  | |  |
| URRBMI | 56 (87.5) | 6 (100) | 34 (89.5) |  |  | 49 (86) | | 18 (90) | | 29 (93.5) | |  | |  | | 49 (84.5) | | 18 (94.7) | | 29 (93.5) | |  | |  | | 39 (88.6) | | 9 (81.8) | | 48 (90.6) | |  | |  |
| Self-financed | 1 (1.6) | 0 (0) | 1 (2.6) |  |  | 2 (3.5) | | 0 (0) | | 0 (0) | |  | |  | | 1 (1.7) | | 0 (0) | | 1 (3.2) | |  | |  | | 1 (2.3) | | 0 (0) | | 1 (1.9) | |  | |  |

**Notes:** ^a^: chi-square tests，^b^: Fisher’s exact test

Stable: profile1 to profile 1, profile2 to profile 2, profile3 to profile 3; Unfavorable transition: profile1 to profile 2, profile1 to profile 3, profile2 to profile 3; Favorable transition: profile2 to profile 1, profile3 to profile 1, profile3 to profile 2;

BMI, Body Mass Index, calculated as weight in kilograms divided by height in meters squared; UEBMI, Urban Employee Basic Medical Insurance; URRBMI, Urban and Rural Resident Basic Medical Insurance
